# Innovative epilepsy management: a combined figure of EEG categorization and medication mechanisms

**DOI:** 10.3389/fneur.2025.1534913

**Published:** 2025-01-29

**Authors:** Mohamed Taha, Douglas R. Nordli, Carol Park, Douglas R. Nordli

**Affiliations:** Comer Children’s Hospital, University of Chicago, Chicago, IL, United States

**Keywords:** epilepsy, antiseizure medication, rational pharmacological treatment, EEG, education

## Abstract

**Introduction:**

Epilepsy management requires precision in diagnosis and treatment, particularly when selecting antiseizure medications based on specific epilepsy syndromes. We present an innovative educational tool that integrates EEG categorization with antiseizure medication mechanisms, designed to enhance clinical decision-making in epilepsy management.

**Methods:**

This study evaluated a cohort of neurology trainees through a pre-test and post-test design. Participants were assessed on their ability to diagnose epilepsy syndromes and select appropriate treatments based on EEG findings before and after exposure to the teaching figure. The figure aligns key EEG patterns with specific epilepsy syndromes and outlines the corresponding mechanisms of action of antiseizure medications.

**Results:**

Post-test results demonstrated a statistically significant improvement in trainees’ ability to analyze clinical cases and make informed treatment decisions (mean pre-test score: 52.8; post-test score: 66.5; *p* = 0.0019). The figure facilitated a deeper understanding of the relationship between EEG findings and medication selection, particularly in complex cases.

**Discussion:**

The integration of EEG patterns with antiseizure medication mechanisms allows for more precise epilepsy syndrome diagnosis and enhances the selection of rational polypharmacy approaches. This approach not only improves educational outcomes but also offers potential applications in clinical practice for personalized epilepsy treatment strategies.

**Conclusion:**

This innovative figure bridges the gap between EEG categorization and treatment strategies, providing a valuable tool for improving epilepsy management education and clinical outcomes.

**Plain language summary:**

This manuscript introduces a teaching tool that helps providers better understand how brainwave patterns (EEGs) relate to epilepsy types and guides them in choosing the most effective seizure medications.

## Introduction

Epilepsy management is at the forefront of neurology and epileptology, where treatment precision can significantly impact patient outcomes. To enhance this precision, we present an innovative teaching figure that bridges the gap between EEG categorization and epilepsy syndrome management. This tool simplifies the complex relationship between specific EEG patterns and corresponding epilepsy syndromes while connecting them with tailored treatment options. Grounded in expert opinions and informed by sentinel articles and key literature, the figures provide a comprehensive overview of effective strategies for aligning EEG patterns with appropriate treatments.

## Methods

**Figure 1:**
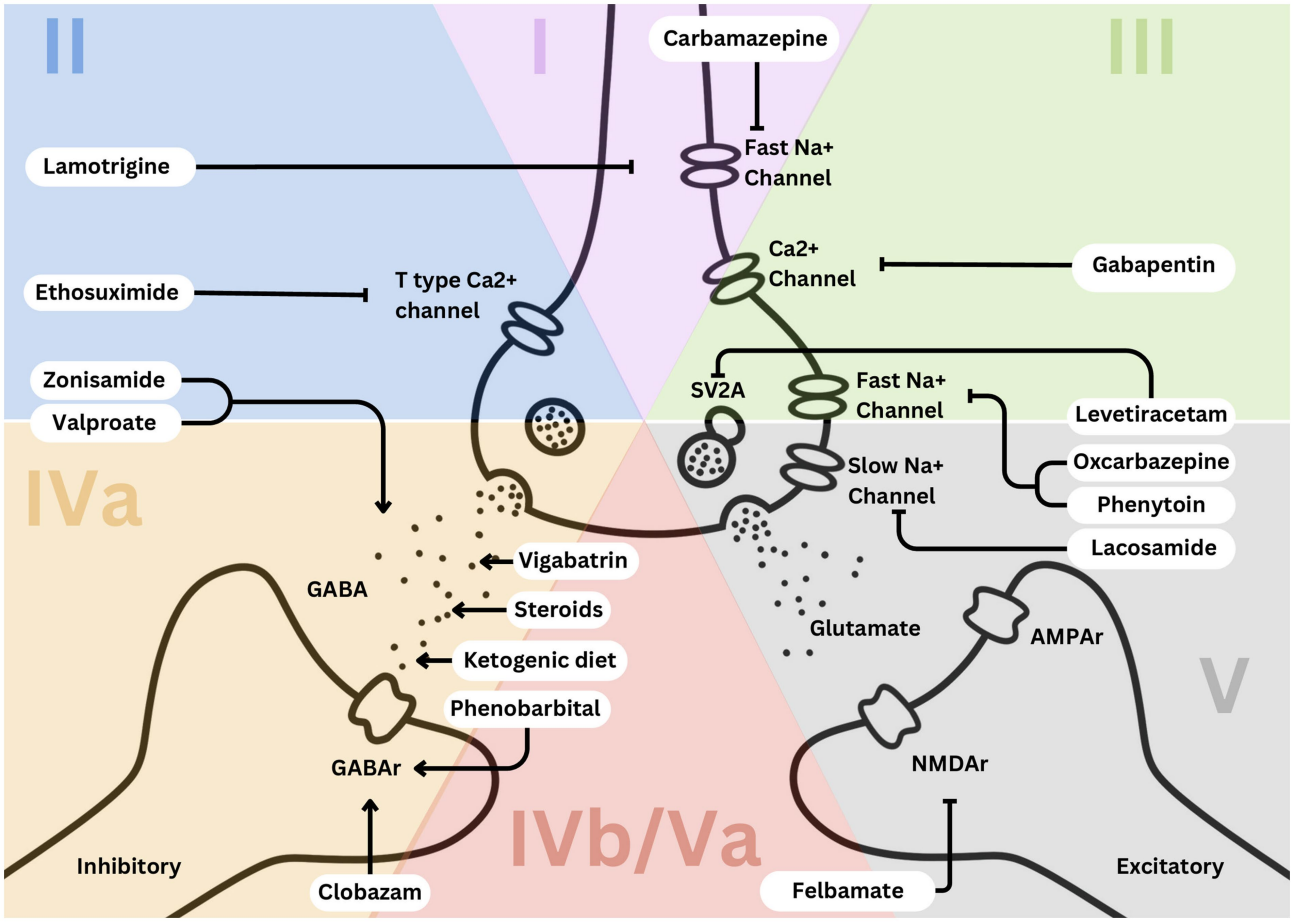
Simplified EEG categorization and predominant mechanisms of action of effective medications leveraged in each category.

**Figure 2:**
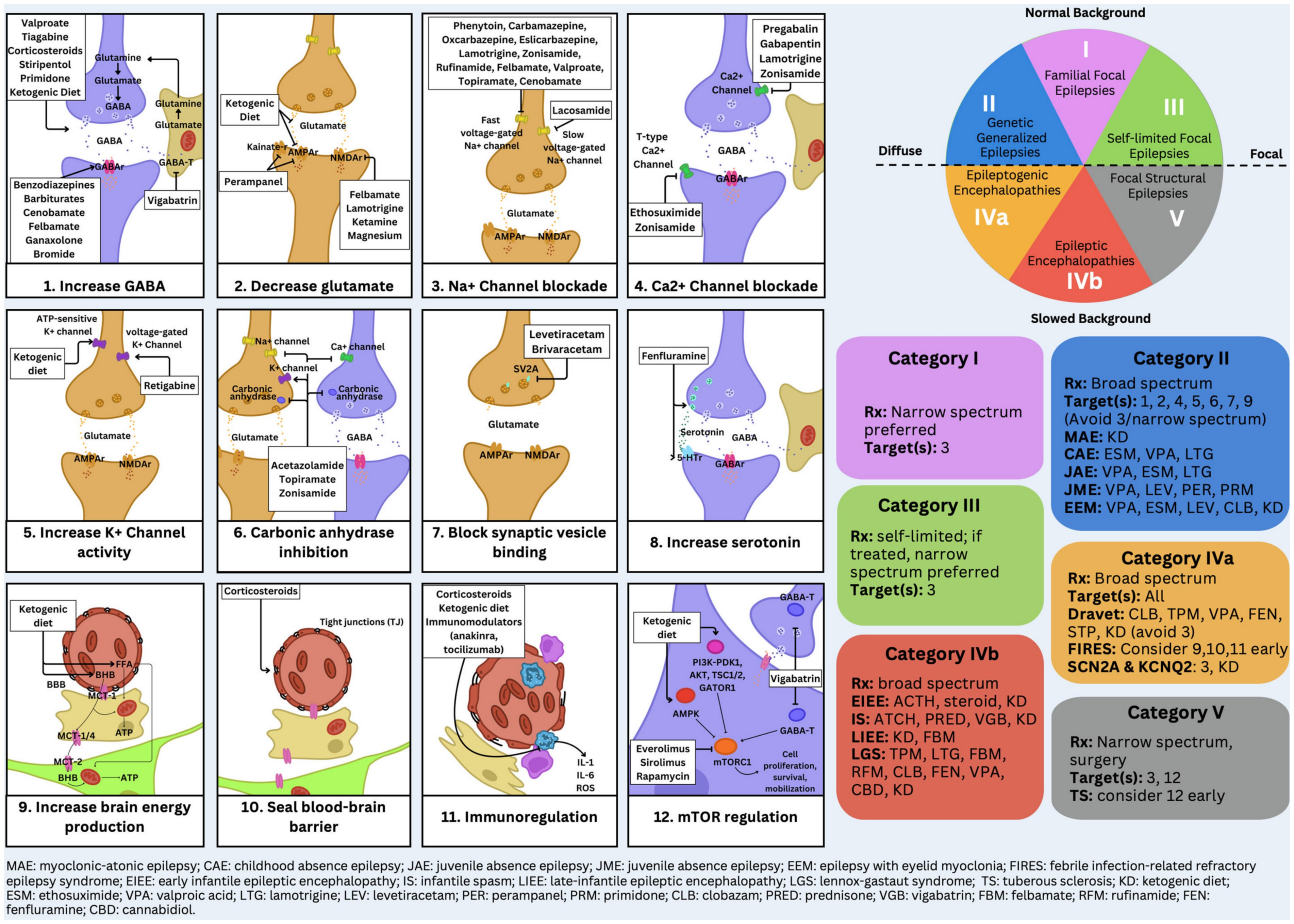
Detailed EEG categorization and mechanism of action figure.

**Figure 3:**
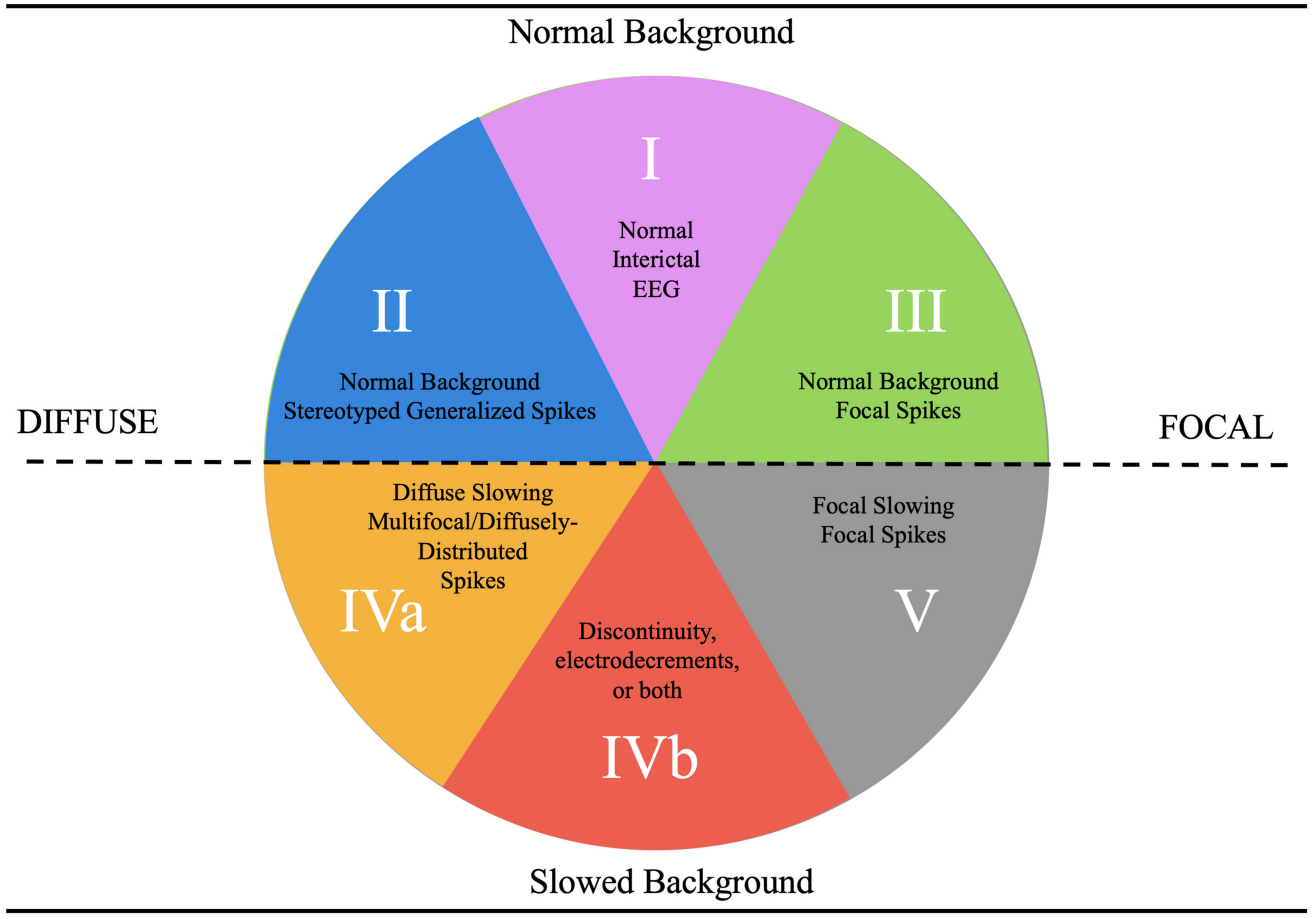
EEG categorization concept figure.

**Figure 4:**
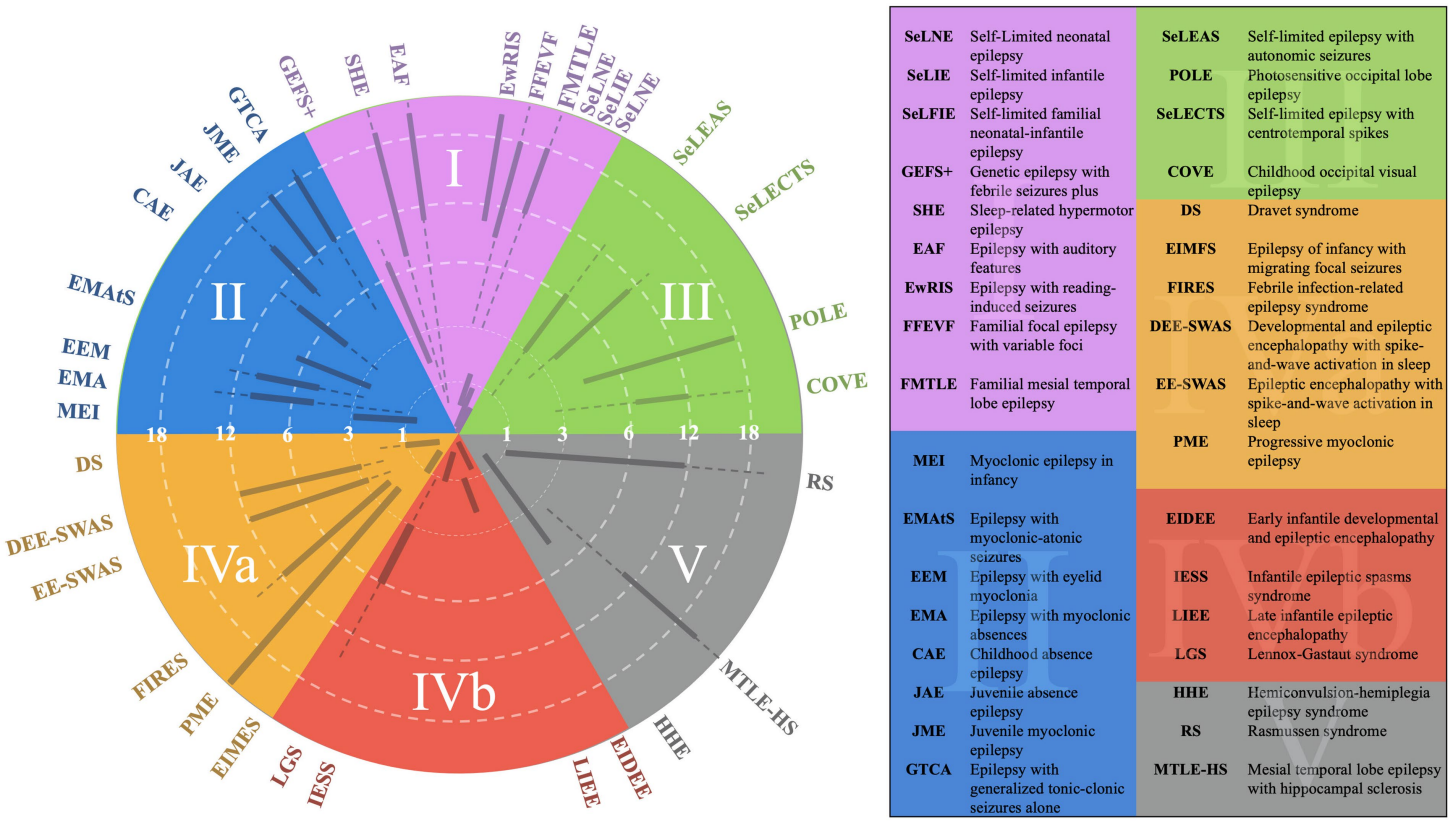
EEG categorization concept figure with superimposed epilepsy syndromes.

We administered a pre-test and post-test to a cohort of 25 trainees to evaluate their ability to diagnose epilepsy syndromes and select appropriate treatments based on EEG findings. The test, designed by two board-certified epileptologists (DRNJr/DRNIII), was conducted during an annual epilepsy oriented educational course attended by child neurology trainees, adult neurology trainees, and researchers. Participants completed the test both before and after attending a lecture that introduced a novel teaching framework, based upon a methodology of understanding epilepsy syndrome through EEG categorization (1, 2). Following the lecture, they were permitted to use Figures 1, 2 to take the test again. The figures aim to connect key EEG patterns (Figure 3) with specific epilepsy syndromes (Figure 4) and outlines the corresponding mechanisms of action of antiseizure medications (Figures 1, 2) (1, 2). The test, available in the Supplementary materials, followed the structured methodology outlined in this manuscript, providing a comprehensive assessment of the trainees’ learning progress. The test was designed to be relevant to multiple epilepsy syndromes and tiers of management.

The set of questions provided covers a broad spectrum of epilepsy-related topics, ensuring a balanced evaluation of fundamental and advanced epilepsy management concepts. A total of 20% of the questions focus on epileptic encephalopathy, requiring high applied knowledge to address complex scenarios, while 10% center on the management of epileptogenic encephalopathy, emphasizing recall of contraindications and mechanisms. Questions related to genetic generalized epilepsy constitute 30% of the total, split between recall and applied knowledge, highlighting both foundational and nuanced aspects of treatment strategies. Similarly, 20% of the questions address self-limited epilepsy, combining moderate recall and applied knowledge to test learners on syndrome-specific management. One question, accounting for 10%, uniquely focuses on the ketogenic diet, a key area that remains a barrier for many practicing neurologists. This structured approach aligns with educational objectives for learners at varying levels of expertise and reinforces critical thinking across different epilepsy syndromes.

## Results

The results demonstrated a statistically significant improvement in the trainees’ ability to apply the figures in managing hypothetical patient cases. The mean score of the pre-test group was 52.8, while the post-test group achieved a higher mean of 66.5. A two-sample *t*-test assuming unequal variances revealed a *t*-statistic of−3.32 and a two-tailed *p*-value of 0.001892, confirming that the difference between the two groups was statistically significant (*p* < 0.05) (Table 1). This suggested that the lecture effectively enhanced the trainees’ ability to use the figures in clinical decision-making. Beyond the quantitative outcomes, the trainees provided overwhelmingly positive feedback, with many requesting laminated copies of the figures for continued reference.

**Table 1:** *T* test of pre-test and post-test groups taking epilepsy treatment mechanism of action quiz.

Simplified test results: pre-test and post-test group comparison	
Metrics	Results
Pre – test group mean	52.8
Post – test group mean	66.5
*t* - statistic	-3.32
*p*-value (Two - Tailed) statistical significance	0.001892 Yes (*p* < 0.05)

## Discussion

Our approach to understanding and making treatment recommendations emphasizes a comprehensive analysis of the patient’s history, physical examination, and EEG findings. This integrated approach is essential for identifying nuances that may not be apparent through history and physical examination alone. For instance, subtle differences between a patient presenting with a severe complex febrile seizure and one with early manifestations of Dravet syndrome can often be clarified by EEG, which may reveal distinct features such as occipital spikes and background slowing (1).

Patient and treatment decisions are always approached with the patient as the primary focus. While the initial analysis is based on the patient’s history and physical examination, evaluating the EEG background is a critical subsequent step (2). This assessment helps determine the alignment of the case within broader diagnostic frameworks, refining the approach to diagnosis and treatment. A careful analysis of interictal epileptiform discharges further hones the diagnostic process, enabling clinicians to make precise treatment decisions. By emphasizing the mechanisms of action of each medication, this approach equips medical professionals to make informed and individualized choices.

To provide clarity on our framework, we will systematically explain each category and discuss the mechanisms of action of the associated seizure medications. These explanations will detail how each medication targets specific neurophysiological processes, offering clear rationales for their use in different epilepsy syndromes.

Category 1 epilepsy syndromes are characterized by a normal EEG background, with a well-defined posterior dominant rhythm and an anterior-to-posterior gradient (2). Any deviations, such as slowed rhythms or disrupted gradients, typically exclude a patient from this category unless an alternative explanation is evident (2). These syndromes include self-limited neonatal epilepsy, generalized epilepsy with febrile seizures plus, epilepsy with auditory features, and familial medial temporal lobe epilepsy (1). They are often associated with infrequent seizures, a strong familial tendency, and high pharmacosensitivity (2).

The primary treatment for Category 1 epilepsy syndromes involves fast sodium channel inhibition, utilizing medications such as carbamazepine and lamotrigine (3). Carbamazepine stabilizes the inactivated state of sodium channels, reducing neuronal excitability and preventing seizure propagation (Figures 1, 2). Lamotrigine inhibits voltage-sensitive sodium channels, decreasing glutamate release and further limiting seizure spread (Figures 1, 2) (4, 5). Neonatal-onset epilepsy, including cases linked to pathogenic variants in the KCNQ2 gene, demonstrates exquisite sensitivity to carbamazepine (5). This effectiveness, highlighted by normal EEG backgrounds and characteristic seizure patterns, underscores the importance of EEG analysis in guiding treatment decisions.

Category 2 epilepsy syndromes also present with a normal EEG background but are characterized by stereotyped generalized spike-and-wave discharges rather than focal spikes (2). Stereotyped discharges exhibit predictable morphology, in contrast to pleomorphic discharges, which are more variable, such as those observed in the “stormy phase” of Dravet syndrome (Supplementary Figure 1) (6). Syndromes within this category include childhood absence epilepsy (CAE) and juvenile myoclonic epilepsy (JME). Myoclonic-astatic epilepsy, formerly known as Doose syndrome, is positioned near the border between Categories 2 and 4a, reflecting its potential for EEG background changes during the stormy phase and its association with monogenic variants that may cause EEG slowing independent of epilepsy (Figure 4) (1). These features often render such conditions resistant to typical Category 2 treatments (6).

Treatment for Category 2 syndromes focuses on T-type calcium channel inhibition and enhancing GABAergic activity (Figures 1, 2). Ethosuximide inhibits T-type calcium channels in the thalamic reticular nucleus, disrupting the thalamocortical loop responsible for generating generalized spike-and-wave discharges (7). Valproate increases inhibitory neurotransmission by enhancing GABA synthesis and inhibiting its degradation (Figures 1, 2). Zonisamide, which blocks sodium and T-type calcium channels and enhances GABA release, is also effective in managing juvenile myoclonic epilepsy (Figures 1, 2) (8, 9).

Category 3 epilepsy syndromes feature a normal EEG background and focal, stereotyped spikes (2). Examples include self-limited epilepsy with autonomic seizures (sELEAS) and self-limited epilepsy with centrotemporal spikes (seLECTS), previously known as Panayiotopoulos syndrome and Rolandic epilepsy, respectively (Figures 3, 4) (10). These syndromes may not require medication, but when treatment is necessary, narrow-spectrum agents targeting fast sodium channels are effective. Gabapentin, particularly beneficial in Rolandic epilepsy, binds to the alpha-2-delta subunit of voltage-gated calcium channels, reducing excitatory neurotransmission and enhancing GABA synthesis (Figures 1, 2) (11). This mechanism modulates neuronal excitability without directly binding to GABA receptors (12). Gabapentin has shown effectiveness in controlling seizures in Rolandic epilepsy and is considered a viable option alongside other antiepileptic drugs such as carbamazepine and levetiracetam (12).

The southern hemisphere of the classification system includes epilepsy categories characterized by EEG background dysfunction, representing more severe epilepsy syndromes compared to those in the northern hemisphere, where the EEG background remains normal (Figure 3). These categories are divided into Categories 5, 4a, and 4b, each defined by distinct EEG features and their implications for epilepsy syndromes (Figure 3).

Category 5, positioned in the far bottom right, is distinguished by focal background slowing and pleomorphic spikes, indicative of localized dysfunction within the brain (Figure 3). The pleomorphic spikes exhibit variability in morphology and timing, reflecting the heterogeneous nature of the underlying pathology. On the opposite side of the spectrum lies Category 4a, described as epileptogenic encephalopathies (Figure 3). These syndromes are marked by diffuse background dysfunction and diffuse pleomorphic epileptiform discharges, suggesting global brain involvement in their pathophysiology. A key distinction between Category 4a and 4b lies in the continuity of the EEG background (Figure 3). Category 4b is characterized by background discontinuity, with electrodecremental periods marked by significant reductions in EEG activity. This feature is typically associated with severe encephalopathy and reflects a higher degree of cortical dysfunction.

Category 4a syndromes, including Dravet syndrome, epileptic encephalopathy with spike–wave activation in sleep (EE-SWAS), and developmental and epileptic encephalopathy with spike–wave activation in sleep (DEE-SWAS), are often linked to monogenic pathogenic variants. These conditions are ideal candidates for precision therapies targeting specific molecular pathways (13). The primary treatment strategy for these syndromes focuses on potentiating GABAergic activity to reduce neuronal excitability and control seizures.

Steroids, such as prednisone, enhance GABAergic transmission by allosterically modulating GABA_A_ receptors to increase their inhibitory effects (Figures 1, 2) (14). Vigabatrin elevates GABA levels by inhibiting GABA transaminase, thereby preventing its degradation (Figures 1, 2) (15). The ketogenic diet induces ketosis, which enhances GABA synthesis and decreases neuronal excitability, making it a valuable option for refractory epilepsy (16). Phenobarbital acts by prolonging the opening of chloride ion channels at GABA_A_ receptors, amplifying neuronal inhibition (Figures 1, 2) (17). Clobazam, a benzodiazepine, increases the frequency of chloride channel opening at the GABA_A_ receptor, further enhancing GABAergic inhibition (Figures 1, 2) (18).

These therapies underscore the importance of targeting GABAergic transmission in managing complex epilepsy syndromes, providing a foundation for effective treatment strategies in both epileptogenic encephalopathies (Category 4a) and epileptic encephalopathies (Category 4b) (14, 15). Category 5 encompasses epilepsy syndromes characterized by focal slowing and pleomorphic epileptiform discharges, including structural epilepsies like medial temporal lobe epilepsy and severe conditions like Rasmussen encephalitis and FIRES (10, 17). Effective seizure control in medial temporal lobe epilepsy often involves sodium channel inhibition, with lacosamide being particularly effective due to its action on slow sodium channels (9).

Felbamate is effective for both Categories 4b and 5 due to its ability to modulate NMDA receptors, which become up regulated in patients with high seizure burdens (Figures 1, 2). It targets heightened NMDA receptor activity when GABA receptors are down regulated during frequent seizures, highlighting its critical role in managing these challenging epilepsy syndromes (5, 19).

## Conclusion

The methodology and figures outlined above proved to be highly effective visual aids, particularly valuable for early-career doctors and clinicians learning about epilepsy management. Our hope is that teaching tools such as these will enhance the understanding of epilepsy management and promote rational, evidence-based approaches to patient care. Further, this approach encourages us to explore novel strategies to mitigate seizures and, ultimately, to discover mechanisms that could lead to a cure for epilepsy.

### Limitations and future directions

Despite the promising results, this study has limitations that must be addressed. The relatively small sample size may limit the generalizability of the findings, and the lack of objective long-term feedback precludes a thorough evaluation of the teaching figure’s sustained impact on clinical practice.

Future research should involve larger, more diverse cohorts and incorporate longitudinal studies to assess the retention of knowledge and the practical application of the figure in real-world settings. Additionally, integrating advanced technologies such as interactive digital tools could enhance the educational impact of the figure. A figure directly commenting on the most efficacious medications specific for seizure types, and not epilepsy syndromes may also be beneficial, as this is a common source of questions from trainees anecdotally.

Finally, further exploration of how this approach can be adapted to accommodate emerging epilepsy treatments and evolving EEG classification systems will be crucial for maintaining its relevance in the dynamic field of epilepsy management.

## Data Availability

The raw data supporting the conclusions of this article will be made available by the authors, without undue reservation.
