# Supplementary material for: Innovative epilepsy management: a combined figure of EEG categorization and medication mechanisms
Source: Front Neurol. 2025 Jan 29;16:1534913. doi: 10.3389/fneur.2025.1534913 (PMC11816356; doi:10.3389/fneur.2025.1534913)
Supplement: Supplementary file 2 [file Data_Sheet_1.PDF]

1. A combination of which of the following treatment would be most efficacious to control seizures associated with the EEG shown below in a 2-year-old boy with SCN2A pathogenic variant?

A. Depakote and Lamotrigine
B. Levetiracetam and Lacosamide
C. Steroids and Ketogenic diet
D. Vigabitrin and Phenytoin
E. Phenobarbital and Ethosuximide

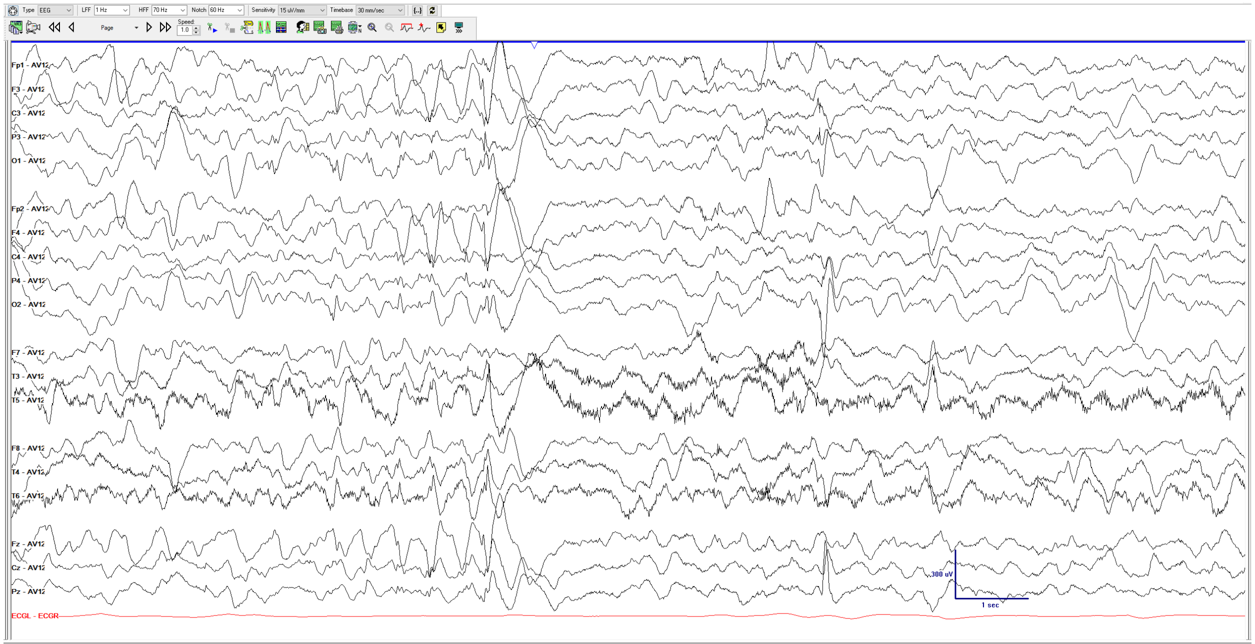

2. A 7-year-old boy from Europe presents to establish care. He currently complains of frequent eye fluttering episodes and unresponsiveness. His EEG is below. In Germany, he was prescribed methsuximide and experienced a severe rash requiring hospitalization. What treatment choice should be offered next?

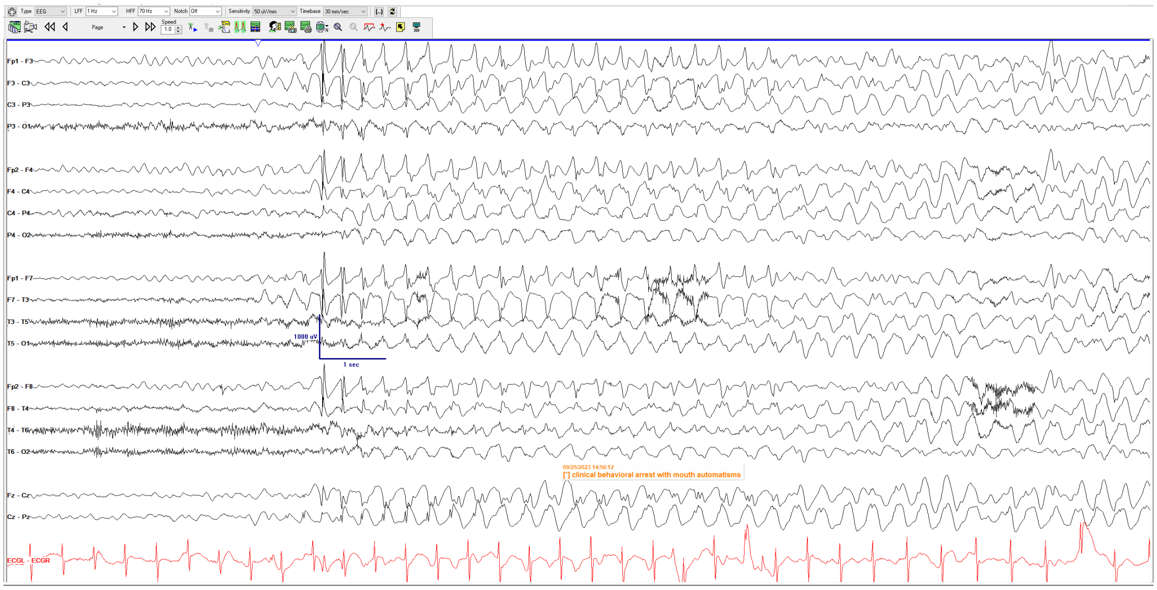

A. Low dose ethosuximide with careful observation and dermatologic consultation
B. Valproic Acid
C. Perampanel
D. Steroids
E. Lacosamide

3. A 15-day old baby girl presents with focal seizures beginning at age 3 days. An epilepsy gene panel reveals a pathogenic variant in KCNQ2. An EEG performed on the same day of visit is unremarkable. There is a strong family history of neonatal seizures. Which of the following agents would be most indicated to prescribe?

A. Lacosamide
B. Levetiracetam
C. Phenytoin
D. Lamotrigine
E. Carbamazepine

4. A 7-year-old girl presents to your clinic with refractory epilepsy. The etiology is determined to be secondary to a large stroke the patient suffered in utero. As an infant the patient suffered from refractory infantile spasms. The patient now has atonic, tonic, generalized and atypical absence seizures. The patient's EEG reveals a diffusely slowed background, pleomorphic epileptiform discharges that are predominantly left hemispheric, paroxysmal fast activity and periods of electrodecrement during wakefulness and sleep. The following treatments have failed to impact the patient's epilepsy: steroids, ketogenic diet, phenobarbital, vigabatrin, clobazam, lacosamide, trileptal, levetiracetam and valproic acid. Parents are interested in trying a new

medication. Which of the following mechanisms of action would be reasonable to pursue in the next treatment trial?

A. Fast sodium channel blockade
B. T –type calcium channel blockade
C. AMPA receptor blockage
D. NMDA receptor blockade
E. SV2A synaptic binding blockage

5. A 32-year-old woman with juvenile myoclonic epilepsy presents to your clinic. Her seizures began at age 14. She was initially treated with lamotrigine which significantly worsened myoclonus, rendering her unable to perform schoolwork. She then trialed levetiracetam but was hospitalized with a major depressive episode. She currently is taking valproic acid but is interested in starting her family with her husband. Which of the following medications presents a reasonable and effective treatment alternative moving forward?

A. Zonisamide
B. Carbamazepine
C. Clobazam
D. Gabapentin

6. Which of the following EEG features indicates an epilepsy syndrome most responsive to treatment with ethosuximide?

A. 3-hz spike and wave discharges in a patient with migraines
B. 3-hz spike and wave discharges in a patient with slowed background for age and developmental delay
C. 3- hz spike and wave discharges in a patient with hyperventilation associated absence seizures.
D. 3- hz spike and wave discharges in a patient with photic stimulation induced absence seizures and eyelid myoclonia.

7. Treatment with what agent in a 1 year old girl with hemiconvulsive seizures associated with fever and hyperthermia would be relatively contraindicated?

   A. Valproic Acid
   B. Zonisamide
   C. Clobazam

D. Oxcarbazepine
E. Felbamate

8. Treatment options for a childhood with panyiatopolous syndrome include which of the following agents?

A. Valproic acid
B. Clobazam
C. Levetiracetam
D. Oxcarbazepine
E. A and B
F. A and C
G. B and C
H. B and D
I. C and D

9. One of the ketogenic diets predominant mechanisms of action is which of the following:

A. GABA potentiation
B. T- type calcium channel inhibition
C. NMDA receptor blockade
D. Glutamate re-uptake inhibition
E. Slow sodium channel blockade

10. True or False: modern studies indicate that self-limited epilepsy syndromes respond better to slow sodium blockade versus fast sodium channel blockage.

A. True
B. False
